# Neutrophilic erythrophagocytosis in myelodysplastic syndrome and cold agglutinin disease co‐occurrence

**DOI:** 10.1002/ccr3.6828

**Published:** 2023-01-11

**Authors:** Asra Amelirad, Parisa Modaresi, Hassan Soltani

**Affiliations:** ^1^ Kowsar Medical Laboratory, Kowsar Hospital Kurdistan University of Medical Sciences Sanandaj Iran; ^2^ Department of Pathology, Kowsar Hospital Kurdistan University of Medical Sciences Sanandaj Iran; ^3^ Public Health Laboratory Group Kurdistan University of Medical Sciences Sanandaj Iran

**Keywords:** anemia, autoimmune, cold agglutinin disease, hemolytic, myelodysplastic syndromes, phagocytosis

## Abstract

In this article, we report the rare phenomenon of erythrophagocytosis by neutrophils in peripheral blood smear of a 56‐year‐old male patient with concurrent myelodysplastic syndrome and cold agglutinin disease.

## Graphical abstract

In this paper, we report a rare case with several notable features. coexistence of MDS and CAD and observation of phagocytized red blood cell precursor nuclei are the most remarkable aspects of this case.

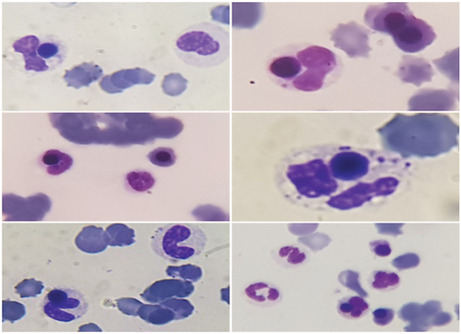

## 1 INTRODUCTION

Myelodysplastic syndromes (MDS) are clonal hematologic malignancies characterized by the bone marrow ineffective hematopoiesis leading to peripheral blood cytopenia.^1^ MDS are frequently associated with autoimmune disease and inflammatory response of the immune system.^2^, ^3^ Some patients with MDS have antibodies against red blood cells (RBCs).^4^ Cold agglutinin disease (CAD), a special type of autoimmune hemolytic anemia (AIHA) caused by auto‐antibodies attacking RBCs in cold temperatures, has infrequently been reported in patients with MDS.^3^, ^5^, ^6^ Neutrophilic erythrophagocytosis phenomenon, in which RBCs are engulfed by neutrophils, is another unusual observation reported in MDS. Underlying mechanism of erythrophagocytosis by neutrophils is still unknown, however, the CR1 receptor of neutrophils can react with C3b bound to RBC.^7^, ^8^, ^9^ Here, we report erythrophagocytosis in a patient with concurrent MDS and CAD.

## 2 CASE PRESENTATION

**FIGURE 1:**
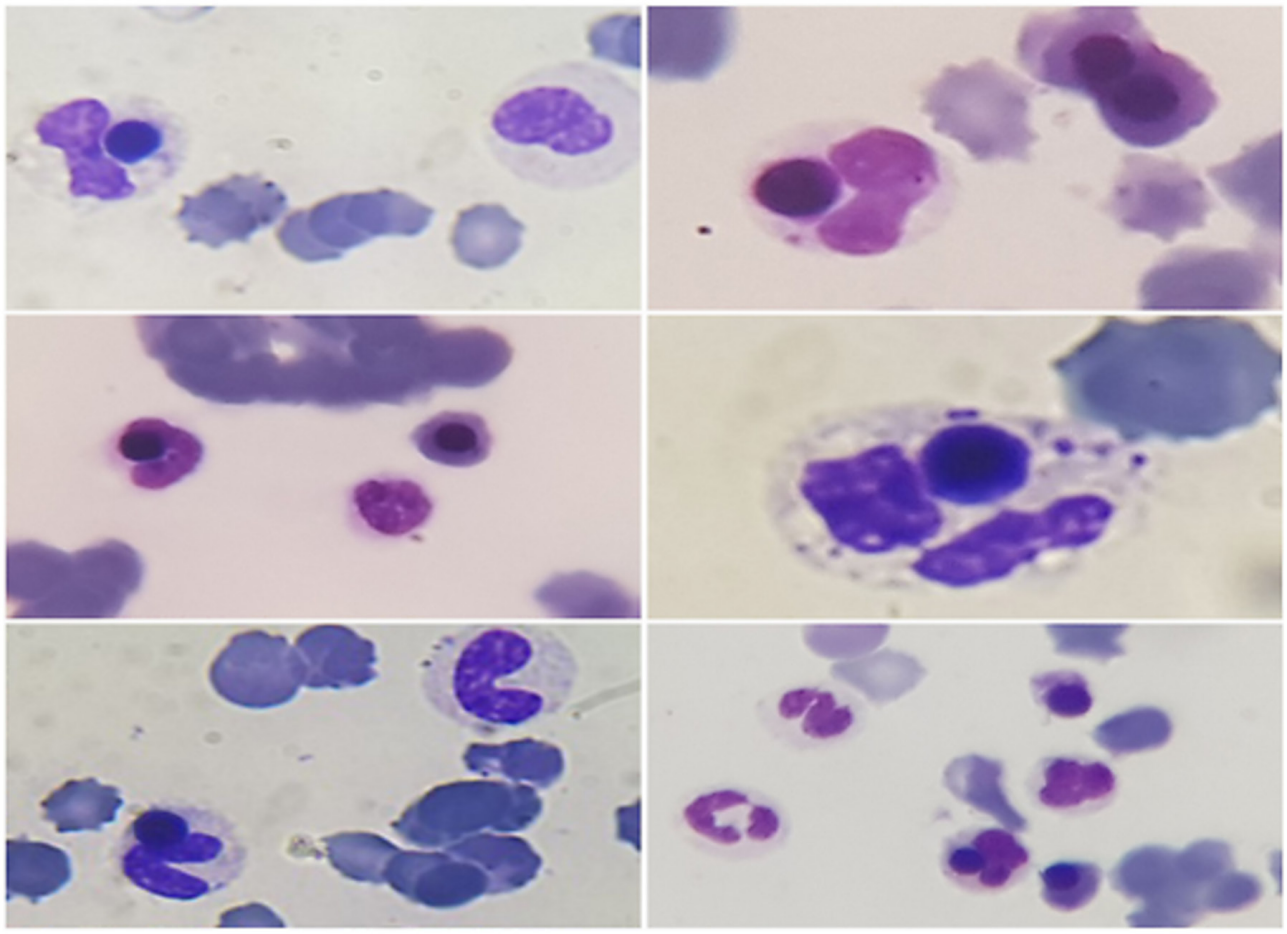
Neutrophils with phagocytized erythrocyte precursor's nuclei in peripheral blood smear of a patient with MDS.

**FIGURE 2:**
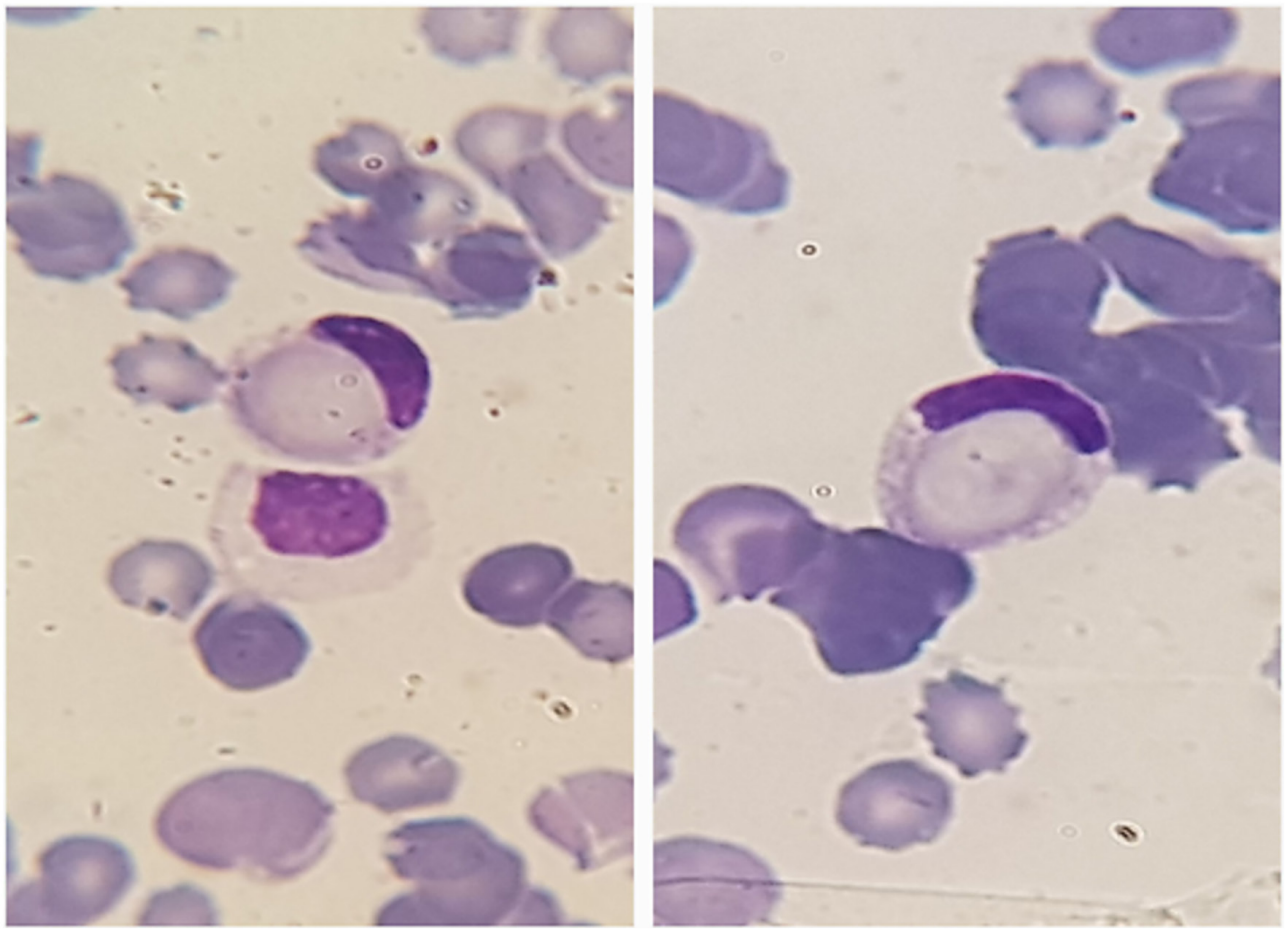
Erythrophagocytosis by neutrophils in peripheral blood smear of a 56‐year‐old male patient with myelodysplastic syndrome.

A 56‐year‐old male patient with severe anemia and low level of consciousness was admitted to the emergency department. The patient having a history of anemia, thrombocytopenia, and RBC agglutination was recently diagnosed with myelodysplastic syndrome with multilineage dysplasia (MDS‐MLD) with 3% blast cells based on bone marrow examination and had not yet received medication to treat MDS. Complete blood count showed severe anemia (2.8 g/dl) and moderate thrombocytopenia (50 × 10^3^/mm^3^). Peripheral blood smear examination showed hyposegmentation in neutrophils, high nucleated red blood cell count, mild RBC agglutination, and erythrophagocytosis in about 2% of neutrophils (Figures 1 and 2). Other laboratory results indicated the following: increased LDH (2536 IU/L), increased total bilirubin (9.31 mg/dl), increased direct bilirubin (3.97 mg/dl), elevated ferritin (>2000 ng/ml), positive direct Coombs (IgG−/C3+), and anti‐i (1:128). After eliminating the blood group discrepancy due to the presence of cold agglutinins and successfully performing cross‐match with preheated serum, two units of pack cells were transfused to this patient. Packed cells were transfused slowly through a blood warmer and the patient was kept in warm condition. However, the patient's condition deteriorated rapidly and he expired 10 h after the blood transfusion due to cardiac arrest.

## 3 DISCUSSION AND CONCLUSION

This case has several noteworthy aspects. The first is the coexistence of MDS and CAD, which is rarely reported. The second is the observation of erythrophagocytosis by neutrophils in the peripheral blood smear, which is a rare morphological phenomenon. The next notable and distinctive aspect of this case is the observation of a large number of phagocytized red blood cell precursor nuclei, which according to the best of our knowledge has not been reported in peripheral blood smear of these patients. We hope this case aid researchers with better understanding of MDS and CAD co‐occurrence.

## AUTHOR CONTRIBUTIONS

**Asra Amelirad:** Investigation; supervision; writing – original draft. **Parisa Modaresi:** Validation. **Hassan Soltani:** Writing – review and editing.

## FUNDING INFORMATION

There is no funding support in this study.

## CONFLICT OF INTEREST

Author have no conflict of interest to declare.

## ETHICAL APPROVAL AND CONSENT

Due to the patient's death, the patient's consent form was completed by his brother and sent to the ethics committee of the Kurdistan University of Medical Sciences, after which the ethics approval code was received. The link to the approval letter of the ethics committee: https://ethics.research.ac.ir/ProposalCertificateEn.php?id=256283.

## Data Availability

All data generated during this study are included in this published article.
